# Evaluation of the Ideal Horizontal X-Ray Beam Angulation to Accurately Identify Two Separate Canals in Maxillary First Premolars—A Retrospective Clinical Study Using Cone-Beam Computed Tomography in an Austrian Subpopulation

**DOI:** 10.3390/dj13040151

**Published:** 2025-03-30

**Authors:** Benedikt Schneider, Luisa Klinkhamels, Wilhelm Frank, Constantin von See, Jörg Philipp Tchorz

**Affiliations:** 1 Center for Oral and Maxillofacial Surgery, Department of Dentistry, Faculty of Medicine and Dentistry, Danube Private University, 3500 Krems, Austria; 2 Division for Endodontics, Center for Operative Dentistry and Periodontology, Department of Dentistry, Faculty of Medicine and Dentistry, Danube Private University, 3500 Krems, Austria; 3 Department of Health System Research, Faculty of Medicine and Dentistry, Danube Private University, 3500 Krems, Austria; 4 Research Center for Digital Technologies in Dentistry and CAD/CAM, Department of Dentistry, Faculty of Medicine and Dentistry, Danube Private University, 3500 Krems, Austria; constantin.see@dp-uni.ac.at

**Keywords:** beam angulation, intraoral radiographs, maxillary, premolar, tube shift, X-ray

## Abstract

**Background/Objectives:** Intraoral (IO) radiographs are critical for endodontic diagnostics, yet conventional orthoradial imaging often results in superimposition, limiting the visibility of individual root canals. Maxillary first premolars pose challenges due to their anatomical characteristics and positioning within the dental arch. This study aimed to retrospectively analyze cone-beam computed tomography (CBCT) data to determine the horizontal X-ray beam angulations for maxillary first premolars at which root canals overlap and neighboring tooth superimposition occur, providing clinically relevant guidance for optimizing IO radiographic techniques. **Methods:** CBCT scans from 85 patients were analyzed using ImageJ software to measure the angles at which maxillary first premolar root canals overlap or become obscured by adjacent teeth. The mean angles for canal overlap and neighboring tooth superimposition were determined. Statistical analysis was performed using SPSS Version 29.0, and the level of significance was set to 5%. **Results:** The mean angle for root canal overlap was 93.56° (±10.08). The angles at which neighboring teeth began to superimpose were 124.38° (±9.91) for the distal contour of the canine and 63.46° (±9.38) for the mesial root contour of the second premolar. No significant differences were observed between apical and coronal measurements for root canal overlap but tapering of the roots led to significant differences in neighboring tooth superimposition (*p* < 0.05). **Conclusions:** A mesial beam shift within a calculated safe corridor (98.5–129.5°) optimizes canal separation without superimposition from adjacent teeth. For ideal visualization, a mesial angulation close to 40° is recommended. These findings support improved IO radiographic techniques while minimizing the risk of retakes in adherence to the ALARA principle.

## 1. Introduction

Intraoral (IO) radiographs are an indispensable diagnostic tool in dentistry, playing a critical role in the assessment of periapical pathologies, visualization of surrounding anatomical structures, and evaluation of root canal morphology [1,2,3]. In endodontics, IO radiographs are essential for verifying working length, confirming master cone fit, assessing the quality of obturation, and follow-ups [4,5,6,7,8]. However, their effectiveness hinges on the ability to distinguish individual roots and their apices clearly, as the precision of chemo-mechanical disinfection within root canals significantly impacts treatment outcomes [9]. A key limitation of traditional orthoradial (right-angled) radiographs lies in their two-dimensional nature, which often results in superimpositions and obscures critical details, particularly in teeth with complex anatomy such as multiple canals within a single root or multi-rooted teeth [6,8,10]. To overcome these challenges, eccentric radiographs—achieved by horizontally shifting the X-ray beam mesially or distally—have proven indispensable for enhancing diagnostic accuracy [6,8,11,12].

Maxillary first premolars present a unique set of challenges in both endodontic treatment and radiographic imaging due to their complex anatomy and geometric positioning within the dental arch. Factors such as the curvature of the anterior palate, proximity to neighboring teeth, and variations in root canal configuration often complicate the acquisition of optimal IO radiographs [13]. Clinically, this can lead to repeated radiographs at different angulations, increasing radiation exposure, and deviating from the ALARA (As Low As Reasonably Achievable) principle. To minimize these errors and reduce unnecessary radiation, it is crucial to optimize imaging techniques from the outset.

Several studies have explored the efficacy of varying horizontal X-ray beam angulations to improve the identification of root canals oriented in a bucco-oral direction. Most studies in this area have relied on a trial-and-error approach, employing predefined horizontal angulations ranging from 0° to 40° [12,14,15,16,17]. For instance, Karnasuta et al. [12] investigated the impact of varying horizontal beam angulations on the radiographic separation of superimposed canals in maxillary premolars. Their findings indicated that higher degrees of horizontal tube shift increased the likelihood of canal separation. However, since their study was conducted in an ex-vivo setting, the clinical applicability of these results remains limited and should be interpreted with caution.

To address this gap, the aim of this study was to measure actual angles in vivo, instead of adopting a trial-and-error approach, by retrospectively analyzing cone-beam computed tomography (CBCT) data to identify specific eccentric angles at which two individual root canals in maxillary first premolars overlap, both coronally and apically, or the outer root surface gets superimposed by neighboring teeth. This approach seeks to provide clinically relevant insights that can enhance diagnostic accuracy and reduce the need for repeated radiographs in endodontic practice.

## 2. Materials and Methods

For this retrospective analysis, cone beam computed tomography (CBCT) scans of one hundred patients were randomly chosen from the university’s clinical database. The patient population resulted from the geographical area covered by the clinic. All CBCTs were obtained using a Dentsply Sirona Orthophos SL 3D imaging (Dentsply Sirona, Bensheim Germany) unit tube voltage: 60–90 kVp; tube current: 3–16 mA. The selected volumes were either 8 × 8 cm or 11 × 10 cm with standard or high-definition resolution. All CBCTs had to be free of any significant distortions or recording errors and of good image quality. Low-dose CBCTs or smaller volumes than 8 × 8 were not chosen for the evaluation. All CBCTs were taken either for implant planning or prior to surgeries, such as impacted third molar removal, orthodontics, or other diagnostic reasons. None of the selected CBCTs were specifically enrolled for this study, and no patients were exposed to additional radiation. All patient data were pseudonymized according to local data protection regulations to protect patient confidentiality. Written informed consent was obtained from all participants. Our study protocol was approved by the Institutional Ethics Committee (DPU-EK/038; Approved on 11 January 2024).

The sample size of 100 CBCT scans was determined based on previous studies evaluating radiographic techniques and anatomical visualization in maxillary premolars to ensure comparability with existing literature [12,17,18].

### 2.1. CBCT Alignment

**Figure 1:**
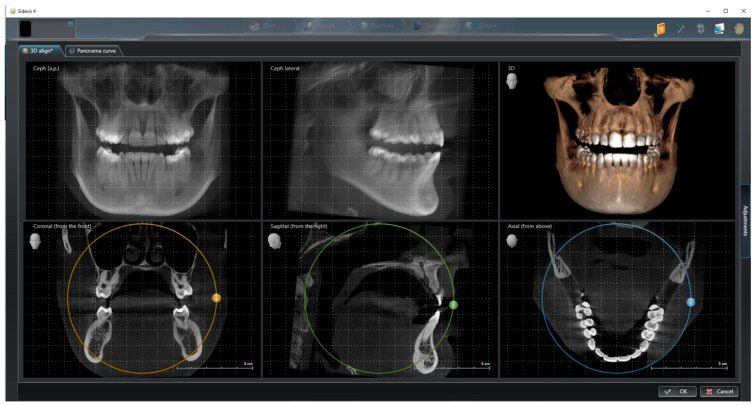
CBCT alignment to correct patient position by adjusting the tilt and rotation.

In the first step, the DICOM data of each patient were opened using SIDEXIS (version 4.3, Dentsply Sirona, Bensheim, Germany), and the patient position was corrected by adjusting the tilt and rotation. For this purpose, the coronal, sagittal, and axial sectional images were aligned parallel to the horizontal and transverse axes by shifting the drag points (Figure 1). The alignment of the patient’s position was necessary to ensure that all subsequent measurements took place in a correct horizontal plane.

### 2.2. CBCT Screenshots

In the next step, the multiplanar reconstruction was used to identify axial planes in which the root canals were clearly visible. The centerline of the maxilla was marked and the coronal screenshot was taken just apically of the pulpal floor. The apical screenshot was taken in the most apical plane where the root(s) were recognizable.

### 2.3. Measurements

**Figure 2:**
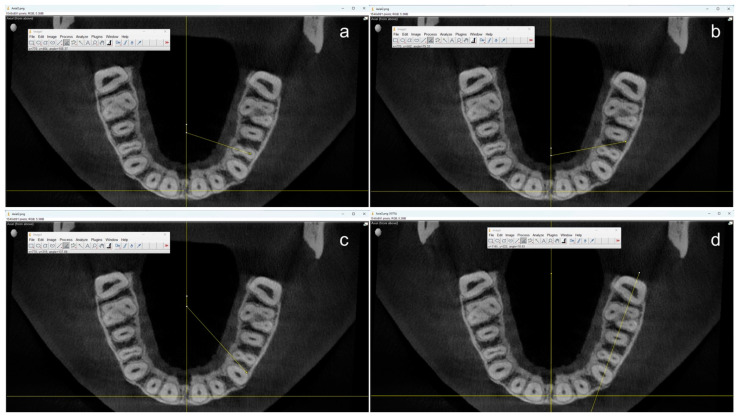
Example of measurements in coronal axial planes using ImageJ. The angle through the root canal(s) (**a**) and the angles where the neighboring teeth would start to overlap the first premolar from the distal (**b**) and the mesial (**c**) were measured using the center of the maxilla as a reference. Additionally, the angle between the jaw and the central axis (**d**) was measured for final calculations.

All measurements were performed by one single operator using the open-source software ImageJ (OpenSource, Version 1.54f) starting from the centerline to ensure a uniform reference position (Figure 2).

First, the angle at which the two root canals of the maxillary first premolars overlap was measured by drawing a line through the center of these canals. Two additional angles were measured to determine the angle at which the neighboring teeth would start to overlap the first premolar. On the one hand, the angle between the distal contour of the adjoining canine and the mesiopalatal contour of the first premolar. On the other hand, the angle between the distal root contour of the first premolar and the mesial root contour of the second premolar. If a root canal was not clearly visible in the apical section, the center of the root contour was used as an orientation aid. Teeth with root canal fillings, root resorptions, atypical anatomy (e.g., more than two roots), and CBCT artifacts to the extent that made valid measurements impossible were not taken into account for this study.

Because it can be assumed that an IO X-ray film or sensor is usually placed parallel to the jaw and not at the center of the palate, the angle between the jaw and the central was additionally measured, and the respective angles were calculated accordingly.

### 2.4. Statistical Analysis

For a descriptive analysis, means and standard deviations were computed. Data were evaluated for normal distribution using the Kolmogorov–Smirnov test. For comparisons between two independent groups, either the Mann–Whitney U test or an independent t-test was applied, depending on the distribution of the data. All calculations were performed with the statistical software SPSS Version 29.0 (IBM, Armonk, NY, USA), and the level of significance was set to 5%.

## 3. Results

Out of the 100 CBCT cans initially selected, final measurements were conducted on 85 patients. These included 33 males and 52 females. Due to factors such as missing maxillary first premolars, missing neighboring teeth, and/or small field of views (e.g., 8 × 8 cm), the final sample consisted of 81 maxillary right first premolars and 80 maxillary left first premolars, which were measured in both apical and coronal axial planes. Out of these maxillary first premolars, 78 had one root, 83 had two roots, nine had a single oval-shaped root canal, and 152 exhibited two separate canals.

The results of the descriptive analysis are presented in Table 1. The overall mean angle at which the root canals of maxillary premolars overlap was 93.56° (±10.08). Additionally, the mean angles at which the distal contour of the adjoining canine and the mesiopalatal contour begin to superimpose, as well as the angle at which the distal root contour of the first premolar and the mesial root contour of the second premolar start to overlap, were 124.38° (±9.91) and 63.46° (±9.38), respectively. No significant differences were observed between apical and coronal measurements for the angles at which root canals overlap. However, the tapering of the roots in the apical region led to significant differences in the angles at which the superimposition of neighboring teeth first occurred (*p* < 0.05). The gender of the patients from whom the CBCT data were obtained did not have a significant influence on measurements.

## 4. Discussion

Dixon and Hildebolt [19] gave an overview of film holders and realized that existing devices have strengths and weaknesses. They concluded that the perfect film holder has still yet to be developed. Commercially available film holders are typically designed to position the X-ray beam at a 90° angle relative to the receptor, aligning it parallel to the axis of the jaw when placed inside the mouth. As a result, any information within the bucco-oral directed beam path is inevitably superimposed. To address this clinical problem, Puapichartdumrong et al. [17] developed a new film holder that enables a horizontal angulation of 20° and 40°. They evaluated its clinical efficacy in comparison to a standard holder and observed that the angulation-adjustable holder resulted in less cone-cutting error and a significant reduction of time for X-ray cone alignment when taking IO radiographs eccentrically. A common approach to evaluating the effect of horizontal X-ray angulation on the visual root canal separation is a trial-and-error principle, which uses predefined angulations [12,14,15,16,17]. To our knowledge, no other study to date has determined specific X-ray beam angles at which root canals in first maxillary premolars overlap or at which neighboring teeth begin to superimpose, using clinical CBCT data.

Other studies used ex-vivo setups with single extracted teeth or reassembled neighboring teeth [11,12,14,15,20]. Such experimental setups may introduce potential sources of error, as the ex-vivo reassembly of extracted teeth or the alignment of a single tooth does not accurately replicate their natural position and angulation within the jaw. Karnasuta et al. [12] evaluated the effect of different horizontal angulations in maxillary premolars with different root morphologies. Regarding two-rooted premolars, canal separation was successful in 100% of the cases when the X-ray was taken at an angle of 15–40 degrees from the mesial. Martínez-Lozano et al. [14] took several radiographs from extracted premolars in horizontal (0°, 20° and 40°) and vertical planes (0°, 15°, 30°) and observed that specifically a horizontal angle of 40° was best for canal visualization.

In contrast to the aforementioned ex-vivo setups, Bardauil et al. [16] performed six IO radiographs using predefined angles in both horizontal (up to 25°) and vertical (15°) directions to evaluate the dissociation quality of maxillary premolar roots in patients. They observed the best diagnostic dissociation when the X-ray beam was shifted horizontally up to the highest angle that was evaluated with an additional vertical inclination of 15°. Based on their observations, they recommended using vertical film holders—typically designed for incisors—for the ideal positioning of intraoral (IO) radiographs of maxillary premolars. However, if the X-ray sensor is positioned upright in the patient’s mouth, there is an increased risk that the tooth may not be fully captured in eccentric X-ray images due to the sensor’s limited width.

To comply with the ALARA principles, we chose to perform measurements in CBCT data retrospectively in order to avoid unnecessary radiation. The advantage of CBCT is that it allows for accurate measurements without any distortion [21,22]. This allowed us to measure the exact angles at which anatomical structures overlapped. However, as CBCTs resolution is normally dependent upon its field of view, and only volumes 8 × 8 cm and larger were chosen, minimal measurement errors due to voxel size and possible artifacts have to be taken into consideration.

Our observations highlight the significant risk of misinterpretation when capturing orthoradial X-rays. Eccentric IO imaging of maxillary first premolars is therefore unavoidable. With the mean angle being 93.56° (±10.08), one could assume that it would be easier to shift the beam towards the distal to distinguish individual root canals more easily. However, anatomical factors, such as the curvature of the anterior palate, limit the clinician’s ability to position an intraoral X-ray receptor sufficiently far anterior to capture eccentric images from the distal direction. Consequently, this approach would only increase the risk of missing the target area. Due to these geometric considerations, an X-ray tube shift toward the mesial is recommended for eccentric IO radiographs of maxillary premolars. By considering the mean angles and standard deviations, a safe corridor can be calculated where the likelihood of individual root canal separation—without superimposition from neighboring teeth—is maximized. The corridor, in this case, lies approximately between 98.5° and 129.5° degrees, which is equivalent to a mesial beam shift of 8.5–39.5 degrees. Since other studies demonstrate that visual canal separation accuracy increases with larger beam shift angles, the mesial beam shift degree should be chosen close to 40° accordingly. The fact that apical and coronal angles were nearly identical indicates that both roots or root canals had a similar curvature. Our results do not allow any conclusion, whether they were straight or severely curved. In rare cases, where only one single root canal is present, or in cases where two root canals merge apically, the aforementioned beam shift will help to visualize root canal morphology when instruments or gutta-percha points are inserted. First, maxillary premolars with three root canals were not evaluated and could be included in future studies. For a clinical implementation of our findings, it is important to take the individual tooth position into account. If a tooth is rotated in the jaw, for example, the ideal beam angle could be completely different. In cases where a CBCT is already available, the individual angle can be easily determined to facilitate intraoral radiography and reduce the risk of unnecessary retakes.

## 5. Conclusions

The results of our study offer a practical guide for choosing the optimal horizontal beam angle in IO X-rays of maxillary first premolars, especially when distinguishing between two root canals, which is essential, such as during endodontic treatment. Given the significant risk of superimposition in conventional right-angled radiographs, shifting the X-ray beam mesially within the calculated safe corridor (98.5° to 129.5°) enhances root canal visualization. For clinicians, this means that A mesial beam shift close to, but not exceeding, 40° from the mesial should be selected to achieve ideal visual canal separation without the superimposition of neighboring teeth. If the tooth is not rotated in the jaw, this approach allows for a high chance of distinguishing two separate canals or, in rare cases, to be sure that only one root canal is present.

## Figures and Tables

**Table 1:** Mean differences and standard deviations associated with varying angles in degree [°] where the root canal(s) of a maxillary first premolar overlap or where the neighboring teeth begin to superimpose. All measurements were taken from a line parallel to the center of the jaw at apical (a) and coronal (c) levels. Apical and coronal measurements were compared within the respective tooth location (right, left).

Location		Angle Through Root Canal(s)		Angle Towards Canine		Angle Towards 2nd Premolar	
	n	(a)	(c)	(a)	(c)	(a)	(c)
right	81	94.96 ± 8.79	93.92 ± 8.91	129.10 ± 8.09	121.35 ± 9.12 ^b^	63.53 ± 9.36	67.52 ± 6.10 ^a^
left	80	91.38 ± 11.35	94.04 ± 10.80	128.14 ± 9.40	119.02 ± 9.18 ^b^	58.58 ± 10.89	64.49 ± 8.26 ^b^

^a^ *p* = 0.004, ^b^ *p* < 0.001.

## Data Availability

The raw data supporting the conclusions of this article will be made available by the authors upon request.
